# Health and eHealth Literacy of Patients With Diabetes in Low-Income Countries: Perspective From Guinea and Burkina Faso

**DOI:** 10.2196/55677

**Published:** 2024-12-03

**Authors:** Ismaila Ouedraogo, Borlli Michel J Some, Roland Benedikter, Gayo Diallo

**Affiliations:** 1 Team Assessing Health in a Digitalizing Real-World Setting Bordeaux Population Health Inserm 1219, University of Bordeaux, 146 rue Léo-Saignat, Bordeaux, 33076, France, 33 5 57 57 95 12; 2 Ecole Supérieure d’Informatique School of informatics, Nazi Boni University, Bobo-Dioulasso, Burkina Faso; 3 Center for Advanced Studies, Eurac Research, Bozen-Bolzano, Italy

**Keywords:** health literacy, eHealth literacy, diabetic patients, Guinea, Burkina Faso, patients with diabetes, diabetes

## Abstract

**Background:**

Diabetes is a significant health concern in sub-Saharan Africa, emphasizing the importance of assessing the health literacy and eHealth skills of hospitalized patients with diabetes. This study evaluated the health literacy and eHealth literacy of patients with diabetes at Donka Hospital in Guinea and Sanou Sourou Hospital in Burkina Faso, providing insights for targeted interventions and mobile health (mHealth) solutions to improve self-management and treatment outcomes.

**Objective:**

The aim of this study is to evaluate the levels of health literacy and eHealth literacy among patients at Sanou Sourou Hospital in Burkina Faso and Donka Hospital in Guinea.

**Methods:**

The study included 45 participants from Donka Hospital and 47 from Sanou Sourou Hospital. Data collection took place in May 2022, focusing on variables such as gender, age, education, income, and technology access. Health literacy and eHealth literacy were measured using the Brief Health Literacy Screen (BHLS) and the eHealth Literacy Scale (eHEALS), respectively. Statistical analysis was performed using SPSS 28.

**Results:**

The results indicated that 64% (64/99) of participants at Donka Hospital and 57% (57/99) at Sanou Sourou Hospital were female. The majority of participants (48/98, 49% in both hospitals) fell within the age range of 25-50 years. High rates of illiteracy were observed (62/100, 62% in Donka Hospital and 59/100, 59% in Sanou Sourou Hospital). Smartphone ownership was prevalent (62/99, 62% at Donka Hospital and 64/100, 64% at Sanou Sourou Hospital). Participants reported occasional use of technology for basic purposes and frequent internet usage for accessing health information. However, a significant proportion of participants demonstrated low health literacy (73/99, 73% at Donka Hospital; 79/101, 78% at Sanou Sourou Hospital) and inadequate eHealth literacy (57/100, 57% at Donka Hospital; 62/100, 62% at Sanou Sourou Hospital). Education was observed to positively correlate with health literacy, while age displayed a moderate negative correlation. Weak correlations were observed between gender, income, and health literacy, but these were not statistically significant. No significant correlation was found between the scores of the BHLS and the eHEALS in either hospital.

**Conclusions:**

This study highlights the importance of targeted educational interventions and mHealth solutions aimed at enhancing health and eHealth literacy among hospitalized patients with diabetes. Addressing both health literacy and eHealth literacy is paramount for improving diabetes management and treatment outcomes in Guinea and Burkina Faso. Targeted interventions and mHealth solutions have the potential to empower patients, enabling their active involvement in health care decisions and ultimately improving overall health outcomes.

## Introduction

### Global and Regional Burden of Diabetes

Diabetes remains a significant global health challenge. In 2021, there were 24 million people in Africa living with diabetes, a number projected to rise to 55 million by 2045 [1]. In Guinea, the prevalence of diabetes in adults was 1.7%, corresponding to 103,700 cases, while in Burkina Faso, the number of adult diabetes cases was 164,400, reflecting a similar prevalence rate [1,2]. Inadequate treatment frequently precipitates complications, including end-stage renal disease and blindness stemming from inadequate control of intermediate risk factors such as blood pressure and cholesterol levels [3,4]. The criticality of health literacy, defined as an individual’s capacity to obtain, comprehend, and utilize health-related information to make informed decisions about their health, is palpable within the realm of diabetes management [5]. It correlates with deficits in diabetes knowledge and self-care, imposing a burden on health care providers [6-8]. Diabetes-related health literacy encompasses patients’ ability to understand and effectively apply medical information—a crucial aspect given the complexity of diabetes care [4,9,10].

Mobile technology is becoming increasingly important in supporting health care, especially in sub-Saharan Africa, where it is widespread. In 2022, there were 489 million mobile subscribers in sub-Saharan Africa, with smartphones accounting for 51% of total connections [11]. Forecasts predict that the number of connections in the region will almost double by 2030, with 4G usage expected to reach 49% of total connections [11]. This significant mobile connectivity is evident in countries such as Burkina Faso, with a mobile phone ownership rate of 52.4% in 2019 [12], and Guinea, where the ownership rate reached 76.8% in 2018 [13]. However, mobile internet usage remains low, with only 25% of the population having access due to barriers such as affordability, low digital skills, and inadequate infrastructure [11].

Telemedicine platforms, including the integration of mobile serious health games, are enhancing patient engagement and education [14]. These platforms, which once primarily targeted rural access to health care [15], are now broadening their scope postpandemic to provide more comprehensive health care services. This broadened scope encompasses the delivery of sophisticated interventions, such as prognostic assessment for COVID-19 treatment. In Kenya, for example, an asynchronous provider-to-provider telemedicine model facilitated the delivery of essential health services during the second year of the pandemic [16]. In addition, Vingroup’s DrAid software quickly identified abnormalities in chest X-rays to aid in COVID-19 prognosis [17].

The concept of eHealth literacy has garnered traction, underscoring the significance of patients’ capacity to seek, comprehend, and assess online health information [18,19]. However, ensuring accessibility and user-friendliness remains challenging, particularly for patients with varying communication skills and digital literacy, particularly in low-resource settings [20-23]. With the continuous digitalization of health care, there is a mounting demand for accessible and intuitive health apps, particularly in the aftermath of the COVID-19 pandemic [24].

Although previous studies have investigated health literacy in Guinea [25] and Burkina Faso [26], to our knowledge, this is the first study to examine both health literacy and digital health literacy among hospitalized patients with diabetes in both countries.

### Background on Health and eHealth Literacy

The concept of health literacy refers to an individual’s capacity to access, comprehend, and apply basic health information for active engagement in health-related decision-making processes. It encompasses a diverse array of skills, including general literacy, numeracy, critical thinking, and information retrieval, all of which are essential for active participation in health care. Studies have shown that deficiencies in health literacy can adversely impact health metrics and outcomes [27]. With the health care system progressively embracing technology, the requisite skills for health literacy have similarly evolved.

Digital health literacy, an essential component of general health literacy, involves assessing health information obtained from electronic sources and applying this knowledge to tackle health-related problems. Although digital health literacy shares fundamental aspects with health literacy, it also includes additional skills such as computer literacy, technology literacy, media literacy for navigating search engines, and information literacy for evaluating various sources. Significant differences in digital health literacy and eHealth are particularly evident among demographic groups facing disadvantages in cardiovascular care [28]. Older individuals and those with chronic conditions tend to exhibit lower eHealth literacy [29]. Similarly, individuals with limited education levels are less likely to engage in common eHealth activities, such as monitoring diet and physical activity or communicating with health care providers online [30]. Previous research has shown that racial minorities, such as Black and Latino people, as well as older adults, are significantly less likely to use patient portals, even after accounting for education level [31]. These same demographic cohorts also often encounter challenges with health literacy [27]. Despite the surge in digital interaction within health care, these disparities persist.

In addition, individuals affected by social determinants of health have difficulty accessing eHealth services due to insufficient resources. Although certain groups utilize the internet and smartphones, others, especially older adults and individuals with low incomes, are less likely to possess these technological tools. In addition, understanding digital health content often requires a high level of general education beyond the recommended reading level for medical educational material [32,33]. Complex medical terminology, specialized jargon, dense formatting, and technical language pose significant barriers for people with limited health literacy [34]. Presenting health information in a digital format introduces additional challenges, such as website complexity, navigational difficulties, and the effort required to access web-based health services or apps [32,35]. A survey revealed that nearly half of the people who discontinued mobile health (mHealth) apps cited the tedious data entry or confusion in app usage [20]. Access to health-related internet information, particularly for smartphone users, is critical, especially for underserved communities.

### General Overview of Measurement Tools

With the advent of eHealth technologies, including telemedicine, health apps, and wearable devices, the health care landscape has undergone a substantial transformation. Most notably, these advancements have improved the accessibility of health-related information and facilitated health-related decision-making processes. However, concomitant with these advantages, challenges such as accessibility issues and disparities in technological access have emerged. Subsequently, a plethora of instruments have been devised to assess both health literacy and eHealth literacy, which are pivotal in comprehending individuals’ abilities to effectively navigate and use health information. The Health Literacy Questionnaire by Osborne et al [36] comprehensively evaluates various aspects of health literacy, including the comprehension of health information, navigation of health systems, and social support. The Communicative and Critical Health Literacy Scale [37], introduced in 2013, also contributes to this assessment, as does the Brief Health Literacy Screen (BHLS), a concise clinical instrument [38]. Despite these advancements, research-based health literacy assessment instruments such as the Test of Functional Health Literacy in Adults [39] and the Rapid Estimate of Adult Literacy in Medicine [39] have limitations primarily associated with administration time and protocols [40]. In contrast, instruments such as the BHLS and the Newest Vital Sign offer a quicker, more straightforward assessment of health literacy.

Concurrently, numerous eHealth literacy assessment tools have been developed that focus on individuals’ proficiencies in utilizing digital technologies for health-related purposes. These tools include the eHealth Literacy Scale (eHEALS) [41], developed by Norman and Skinner in 2006, which evaluates an individual’s capacity to access and comprehend health information online. Subsequent instruments, such as the eHealth Literacy Scale [42], have expanded the assessment dimensions to include functional, interactive, and critical eHealth literacy. Furthermore, investigations have explored the interplay among health literacy, numeracy, computer literacy, and internet utilization, using a distinct instrument for each [36-38]. Multidimensional tools such as the eHealth Literacy Questionnaire [43] and initiatives such as the Optimising Health Literacy and Access (Ophelia) process [44] have further contributed to understanding and tackling eHealth literacy challenges.

The extensive utilization of instruments such as eHEALS across diverse studies emphasizes their versatility and reliability in assessing eHealth literacy across diverse populations and languages [45]. These diverse methodologies have enriched our understanding of eHealth literacy and facilitated progress in digital health research and practice.

### The Case of Underserved Communities

Underserved communities in sub-Saharan Africa are confronted with significant health inequalities, characterized by prevalent diseases, limited access to health care, and resource scarcity [46]. The level of health literacy within sub-Saharan Africa remains a critical concern, emphasizing the need for accessible and reliable health information that supports informed decision-making at both the individual and community level. The Agency for Healthcare Quality and Research has addressed this issue in a report on health literacy [47], highlighting the objective measurement of health literacy and its impact on health in many developing countries. A cross-national study on health literacy in sub-Saharan Africa, conducted between 2006 and 2015, covered 14 countries, including Cameroon, the Democratic Republic of Congo, Ethiopia, Ghana, Guinea, Côte d’Ivoire, Lesotho, Rwanda, Niger, Namibia, Sierra Leone, Swaziland, Togo, and Zambia [48]. This study involved 224,751 individuals aged 15-49 years. The prevalence of health literacy was 35.77%, with notable differences between genders and educational levels. Health literacy scores varied significantly, ranging from 8.51% in Niger to 63.89% in Namibia, indicating considerable differences across countries. In addition, Nacanabo et al [26] used the Health Literacy Questionnaire to assess health literacy and its impact on health-related quality of life among patients with type 2 diabetes, suggesting that addressing different health literacy needs could mitigate inequalities and improve the quality of life for individuals with type 2 diabetes. Building upon these antecedent studies, our study aimed to assess the level of health literacy and eHealth literacy among patients with diabetes in hospitals situated in Burkina Faso and Guinea.

## Methods

### Justification of Sample Size and Power Analysis

The sample size was determined using OpenEpi 15 (version 3.01) [49], with a significance level of 95% and a power of 80%. Based on previous research [48], where an expected value of 40% was anticipated for both health literacy and digital health literacy, a risk-prevalence difference of 30% was considered, resulting in a minimum sample size of 88 participants. However, to ensure a better representative sample, 92 participants were ultimately included.

### Settings and Study Participants

Data collection was conducted in May 2022 at Donka Hospital in Guinea and Sanou Sourou Hospital in Burkina Faso. Participants were selected based on eligibility criteria, including a diagnosis of diabetes, age over 18 years (or under 18 years with parental/guardian consent), and proficiency in local languages such as Dioula, Fula, or French.

### Translation of Scales

We used the eHEALS, a widely used questionnaire, to evaluate participants’ digital health literacy [50]. As shown in Table 1, the eHEALS was specifically designed to assess participants’ perceived competencies and confidence in using eHealth information and digital health resources. It serves as a criterion for the suitability of an eHealth-based approach [41] and evaluates skills and knowledge in using eHealth information through 8 items rated on a 5-point Likert scale. These items evaluate the ability to locate, assess, and utilize health-related information from electronic resources, with scores ranging from 8 to 40. Previous studies have distinguished between low eHealth literacy (eHEALS <26) and high eHealth literacy (eHEALS >26) [51].

**Table 1.** eHealth Literacy Scale items and Brief Health Literacy Screen tools.

Items and tools	
**eHealth Literacy Scale**	
Question 1	I know which health resources are available on the internet.
Question 2	I know where to find helpful health resources on the internet.
Question 3	I know how to find helpful health resources on the internet.
Question 4	I know how to use the internet to answer my health questions.
Question 5	I know how to use health information; I can use the health information I find on the internet to help me.
Question 6	I am good at assessing the health insurance companies I find on the internet.
Question 7	I can tell high-quality health resources from low-quality health resources on the internet.
Question 8	I feel confident using information from the internet to form an opinion about my health.
**Brief Health Literacy Screen**	
Question 1	How confident are you in filling in forms yourself? (1=Not at all confident; 2=Somewhat confident; 3=Little confident; 4=Confident; 5=Very confident)
Question 2	How often do you get someone to help you read health information? (1=Not at all; 2=Sometimes; 3=Occasionally; 4=Often; 5=Always)
Question 3	How often do you have problems getting information about your illnesses because of the difficulties you have in reading the health information? (1=Not at all; 2=Sometimes; 3=Occasionally; 4=Frequently; 5=Always)

To assess health literacy, we used the BHLS, a tool renowned for its efficacy in clinical practice and its utility in screening the health literacy of patients with diabetes in resource-limited settings [52,53]. This instrument, which is routinely used in acute care settings, comprises 3 questions on a 5-point Likert scale, aimed at assessing patients’ ability to understand their health status, complete medical forms, and understand hospital materials [54-56]. The BHLS total score ranges from 3-15, with respondents categorized as having low health literacy (total score 3‐9) or adequate health literacy (total score 10‐15) [10,57,58].

As these questionnaires were not validated in the local languages, Dioula and Fula, we started with the translation. Inspired by a previous study by Tenibiaje [59] on the health literacy of ethnic groups in Nigerian prisons, where translation into local languages facilitated participation, we carefully translated the eHEALS and BHLS. This process involved an initial translation followed by a back-translation, which was overseen by an expert committee to ensure accuracy and reliability [60]. Two competent translators in Dioula, employed by the Ministry of Education in Burkina Faso, undertook the translation into Dioula and Fula. Discrepancies between the translations were resolved in a coordination meeting to obtain a standardized version of the questionnaire. The agreement between the translations was evaluated using Cohen κ statistics, resulting in a percentage agreement of 69.23%, which suggests good agreement [61]. Subsequent to the translation process, Cronbach α was computed to evaluate the reliability of the translated questionnaires. This statistical analysis is important for verifying that the items within each questionnaire consistently measure the same underlying construct across different language versions.

### Ethical Considerations

This study was conducted following the principles outlined in the Declaration of Helsinki. Ethical approval was obtained from the Ethics Committee of Souro Sanou University Hospital, Burkina Faso (approved January 28, 2022; number 2022/E 112), and from the Ethics Committee of the National Directorate of Epidemiology and Disease Control, Guinea (approved March 30, 2022; number 246/DNGLEM/MS/2022). Before participating in the study, every participant provided verbal informed consent, demonstrating their voluntary agreement to be involved in the research. Participants were provided with the equivalent of US $1 to cover the cost of a meal. Throughout the research process, strict measures were implemented to ensure the privacy and confidentiality of participant data, safeguarding their rights and well-being.

A verbal declaration of consent was obtained from all participants. For those under the age of 18 years, parental or guardian consent was also obtained, as required by ethical guidelines. Participants were assured of the confidentiality of their data, which was anonymized with unique identifiers to protect their privacy. To respect cultural norms and accommodate participants’ limited literacy skills, verbal consent was preferred over written consent, consistent with the cultural preference for verbal agreements [62]. Throughout the study, participants’ identities were protected, and only identification numbers were used for data management.

### Data Collection Procedure

**Figure 1.**
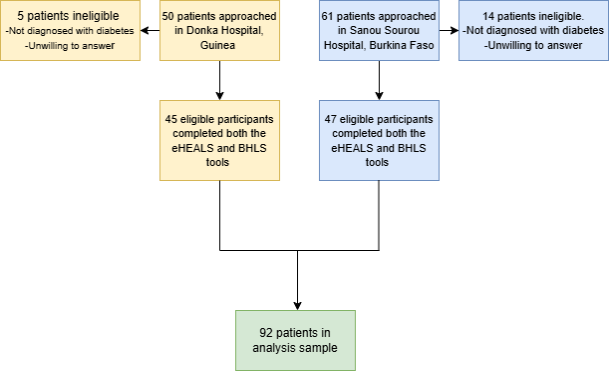
Flowchart of study. BHLS: Brief Health Literacy Screen; eHEALS: eHealth Literacy Scale.

As shown in Figure 1, data collection began with the identification of 92 potential participants at Donka Hospital in Guinea and Sanou Sourou Hospital in Burkina Faso in May 2022. After screening for eligibility based on age, confirmed diagnosis of diabetes, and language proficiency, verbal informed consent was obtained from each participant, emphasizing confidentiality and the right to withdraw. Trained research coordinators conducted structured face-to-face interviews in French. Four trilingual speakers, fluent in Dioula, Fula, and French, administered the translated questionnaires, including the eHEALS and the BHLS. Participants completed both scales during the interviews, guided by clear instructions to ensure the accuracy and honesty of their responses.

### Data Analysis

Data analyses were conducted using IBM SPSS Statistics (version 28; IBM Corp). Descriptive statistics were applied to demographic data, technology use variables, eHEALS scores, and BHLS scores. The internal consistency and reliability of the assessment tools were evaluated using Cronbach α. Multivariate analysis was utilized to explore potential associations between demographic characteristics, health literacy, and eHealth literacy, with statistical significance set at *P*<.05.

## Results

### Demographic Characteristics

Data analysis involved participants who consented to participate. Statistical analyses were conducted using SPSS 28. When comparing the 2 groups, Donka and Sanou Sourou, no statistically significant differences were observed (Table 2). Donka had a slightly higher proportion of women (64/99, 64%) compared to Sanou Sourou (57/99, 57%), although this disparity did not attain statistical significance (*P*=.27). Similarly, both groups demonstrated a comparable age distribution, predominantly falling between 25 and 50 years, with no discernible differences (*P*=.67). In terms of education level, both Donka (62/99, 62%) and Sanou Sourou (60/99, 60%) exhibited a similar proportion of individuals with no formal education or lacking primary school qualifications, with no significant difference observed between the groups (*P*=.62).There was no significant difference in income distribution (P=.71), with respondents earning less than 40,000 CFA francs (US $64), between 40,000–100,000 CFA francs (US $64–$160), or over 100,000 CFA francs (US $161) . Access to technology, as reflected by smartphone ownership, was comparable between Donka (62/99, 62%) and Sanou Sourou (64/99, 64%), with no statistically significant difference observed (*P*=.72). In addition, both groups reported similar patterns of technology use, with no significant difference in use observed (*P*=.72).

To summarize, the analysis shows that Donka and Sanou Sourou have comparable characteristics in terms of gender distribution, age distribution, education level, income distribution, access to technology, and technology use.

**Table 2.** Sample characteristics.

Characteristics and group		Donka Hospital Guinea (n=45), n (%)	Sanou Sourou Hospital Burkina Faso (n=47), n (%)	*t *test	*P* value
**Gender**				1.1116	.27
	Female	29 (64.4)	27 (57.4)		
	Male	16 (35.6)	20 (42.6)		
**Age (years)**				0.577	.67
	<18	2 (4.4)	1 (2.1)		
	18-24	3 (6.7)	2 (4.3)		
	25-49	22 (48.9)	23 (48.9)		
	≥50	18 (40)	21 (44.7)		
**Education**				–0.502	.62
	Uneducated	28 (62.2)	28 (59.6)		
	Primary school	17 (37.8)	19 (40.4)		
**Income**				0.374	.71
	<40,000 CFA (US $64)	23 (51.1)	27 (57.4)		
	Between 40,000 CFA and 100,000 CFA (US $64-$160)	11 (24.4)	13 (27.7)		
	>100,000 CFA (>US $161)	11 (24.4)	7 (14.9)		
**Technology access**				–0.372	.72
	Do not own a cellphone	7 (15.6)	6 (12.8)		
	Own cellphone	10 (22.2)	11 (23.4)		
	Own smartphone	28 (62.2)	30 (63.8)		
**Technology use**				0.368	.72
	Never internet use	16 (35.6)	19 (40.4)		
	Use sometimes for basic tasks (social media such as WhatsApp)	16 (35.6)	20 (42.6)		
	Internet use regularly for information (including health information)	13 (28.9)	8 (17)		

### Internal Consistency and Reliability of Tools

Cronbach α was used to assess the internal consistency and reliability of the eHEALS and BHLS scales in both the Fula and Dioula populations. Normally, a Cronbach α value of .7 or higher is considered satisfactory, while a value exceeding .9 is considered excellent [63,64]. In this investigation, the Cronbach α values for both scales within both populations were determined as follows (Table 3). Specifically, the eHEALS had values of .98 each for both Fula and Dioula, while the BHLS had values of .919 for Fula and .977 for Dioula. These findings denote a notable level of internal consistency and reliability within the scales, indicating correlations between items within each scale and affirming their ability to measure the intended constructs in both populations.

**Table 3.** Cronbach α reliability.

Scales	Cronbach α values, Fula	Cronbach α values, Dioula
eHealth Literacy Scale	.982	.983
Brief Health Literacy Screen	.919	.977

### Participants’ Health Literacy and eHealth Literacy Assessment

At Donka, 73.3% (33/45) of respondents exhibited low health literacy, signifying a deficiency in comprehending and assimilating health information. Conversely, only 35.6% (16/45) demonstrated adequate health literacy, indicating that they are better able to understand and effectively apply health information. Similarly, at Sanou Sourou, 78.7% (37/47) of respondents showed low health literacy, while only 21.3% (10/47) showed adequate health literacy. These findings underscore substantial shortcomings in comprehending and assimilating health information across both hospitals, which emphasizes the necessity for targeted interventions and education. In addition, respondents in both hospitals showed low eHealth literacy, indicating limited mastery of the use of digital technologies for health-related purposes. At Donka, 57.8% (26/45) scored low in eHealth literacy, compared to 26.7% (12/45) who scored high. Similarly, at Sanou Sourou, 61.7% (29/47) had low eHealth literacy, compared to 42.6% (20/47) who had high eHealth literacy. These results emphasize the importance of improving digital health literacy alongside conventional health literacy to ensure the effective use of digital technologies for health purposes in both hospitals.

### Correlations Between Health Literacy, eHealth Literacy, and Demographic Variables

The correlation coefficients presented in Table 4 illustrate the relationships between health literacy and various socioeconomic and demographic factors within the Sanou Sourou and Donka hospitals. In Sanou Sourou, the analysis showed a strong positive correlation between education and health literacy. This indicates that individuals with a higher level of education tend to exhibit higher health literacy scores. The correlation coefficient of 0.94 for education emphasizes the importance of this relationship, and the *P* value of <.001 confirms its validity. This result indicates that promoting education can positively influence health literacy. Of particular interest is the moderately negative correlation between age and health literacy in Sanou Sourou Hospital, with a correlation coefficient of −0.336, indicating that health literacy tends to decrease with age. The *P* value of .02 indicates statistical significance and emphasizes the importance of tailoring health communication strategies to the specific needs of older people.

**Table 4.** Correlation coefficients across health literacy and socioeconomic and demographic variables.

	Gender coefficient	*P* value	Age coefficient	*P* value	Income coefficient	*P* value	Education coefficient	*P* value
**Brief Health Literacy Screen**								
Health literacy mean score, Sanou Sourou Hospital (Burkina Faso)	0.094	.53	−0.336	.02	0.562	<.001	0.944	<.001
Health literacy mean score, Donka Hospital (Guinea)	0.067	.66	−0.286	.06	0.057	.005	0.924	<.001
**eHealth Literacy Scale**								
Health literacy mean score, Sanou Sourou Hospital (Burkina Faso)	−0.276	.06	−0.184	.22	0.407	.004	0.920	<.001
Health literacy mean score, Donka Hospital (Guinea)	−0.102	.50	−0.109	.48	0.417	.42	0.900	<.001

The correlations between gender, income, and health literacy in Sanou Sourou Hospital were weak and not statistically significant. The correlation coefficient for gender of 0.094 indicated a weak positive relationship, but the *P* value of .53 confirmed that this relationship was not statistically significant. Similar patterns were observed at Donka Hospital, where education emerged as the most influential factor positively associated with health literacy, with a correlation coefficient of 0.924 and a highly significant *P* value of <.001. Both hospitals also showed a moderately negative correlation between age and health literacy, although the *P* value for Donka was just above the significance threshold, indicating the need for further research to confirm this relationship.

As for gender and income, the correlations in Donka were weak and not statistically significant, with a coefficient of 0.067 and *P* value of .66. In summary, both Sanou Sourou and Donka emphasized the crucial role of education in improving health literacy. A higher level of education had a strong correlation with better health literacy. Although age exhibited a negative correlation with health literacy, implying that younger people tend to possess higher health literacy, gender and income demonstrated no significant correlations with health literacy in either hospital.

Regarding the relationship between health literacy measured with the eHEALS and the demographic variables, the correlation coefficient between eHEALS and age in Sanou Sourou was −0.184, indicating a weak negative relationship. However, the *P* value of .22 indicates that age may not exert a significant influence on health literacy. In contrast, the correlation coefficient between eHEALS and income was 0.407, indicating a moderately positive relationship, with a significant *P* value of .004, meaning that higher income was associated with greater health literacy.

In Sanou Sourou, the correlation coefficient between eHEALS and education was 0.920, indicating a strong positive relationship, with a *P* value of <.001. The correlation coefficient between eHEALS and gender was −0.276, indicating a weak negative relationship, but the *P* value of .06 indicated that gender did not significantly influence health literacy.

Overall, in Sanou Sourou, education exhibited the strongest positive correlation with health literacy, followed by income, while age and gender exhibited no significant correlations. In Donka, none of the demographic variables analyzed demonstrate a significant correlation with health literacy as measured by the eHEALS, suggesting that age, income, education, and gender do not significantly influence the health literacy of the hospital’s patients.

### Relationship Between BHLS Scores and eHEALS Scores

**Figure 2.**
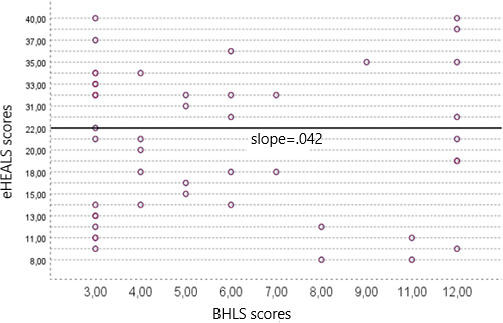
Scatter plot of correlation between eHEALS and BHLS scores at Sanou Sourou Hospital. BHLS: Brief Health Literacy Screen; eHEALS: eHealth Literacy Scale.

**Figure 3.**
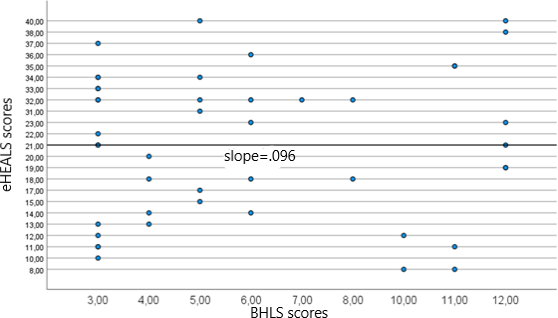
Scatter plot of correlation between eHEALS and BHLS scores at Donka Hospital. BHLS: Brief Health Literacy Screen; eHEALS: eHealth Literacy Scale.

The correlation between the results of BHLS and eHEALS was analyzed using the Pearson correlation. BHLS assesses traditional health literacy and focuses on understanding health conditions, filling out medical forms, and understanding hospital materials. The eHEALS, on the other hand, assesses skills in managing eHealth information and digital technologies. Participants completed both questionnaires and provided a score for each. It is possible for a participant to have low health literacy (as indicated by BHLS score) but high eHealth literacy (as indicated by eHEALS score). This discrepancy results from the different constructs each instrument measures. BHLS assesses traditional health literacy, while eHEALS assesses digital health literacy. Therefore, a participant may encounter difficulty with traditional health materials but demonstrate proficiency in utilizing digital health tools. This discrepancy emphasizes the necessity for a differentiated approach to literacy interventions in both traditional and digital health domains. The correlation analysis between the BHLS and eHEALS scores was conducted in the hospitals of Sanou Sourou and Donka. As shown in Figure 2, in Sanou Sourou, the correlation coefficient was −0.042, indicating a very weak negative relationship, with a nonsignificant *P* value of .78. In Donka Guinea, the correlation coefficient was −0.096, with a *P* value of .53, also without statistical significance (Figure 3).

These results indicate that there was no significant correlation between the BHLS and eHEALS scores at either site. This suggests that these measures of health literacy may capture different aspects and may not correlate consistently within these populations.

## Discussion

### Principal Findings

This study analyses the health and eHealth literacy of patients with diabetes in Donka and Sanou Sourou hospitals. It highlights the critical issue of health literacy in underserved communities, specifically in Burkina Faso and Guinea [47]. The findings reveal significant deficits in patients’ ability to comprehend and use health information, both in traditional and digital formats. Consistent with existing literature, lower educational attainment is associated with lower health literacy levels [65]. This study underscores the importance of educational interventions to enhance literacy in underserved populations. Age was also identified as a key factor, with younger individuals exhibiting higher health literacy [66]. This aligns with previous research showing an age-related decline in health literacy [67]. Tailoring health communication strategies to the needs of older adults can help mitigate this decline and promote better health outcomes.

In contrast to some previous findings [39,68], the study did not reveal significant correlations between gender, income, and health literacy levels. Although gender and income-related differences in health literacy are well-documented, the absence of significant correlations within this study population suggests the necessity for further investigation into the sociocultural factors that influence health literacy in these contexts. The low eHealth literacy observed among patients with diabetes in both hospitals emphasizes their limited competence in using digital technologies for health-related purposes [69]. This observation is consistent with previous research findings highlighting disparities in eHealth literacy, particularly among older people and those with lower levels of education [70]. Addressing these inequalities is crucial to ensuring equitable access to digital health resources and maximizing their benefits for health care delivery. The lack of a significant correlation between scores on the BHLS and eHEALS suggests that these measures may capture distinct facets of health literacy [71]. Although the BHLS focuses on traditional health literacy skills, such as understanding health information and completing medical forms, the eHEALS evaluates competencies in utilizing eHealth resources.

The discrepancy in the correlation between BHLS and eHEALS scores in Guinea and Burkina Faso may be attributed to health information–seeking behaviors influenced by cultural norms [72]. Although reliance on healers, elders, and oral communication for health advice is traditional in these regions [73], digital platforms play a more significant role elsewhere. Furthermore, with the advent of Web 2.0 technologies, renowned for interactivity and user-generated content, there is a revolution in global health information access. However, limited digital literacy and Web 2.0 access in countries such as Burkina Faso and Guinea may pose challenges to conventional health literacy assessments [74]. Therefore, incorporating Norman’s perspective underscores the necessity to reassess eHealth literacy to accommodate these cultural nuances for effective interventions in diverse contexts [74].

Integrating both measures into the health literacy assessment can provide a more comprehensive understanding of individual skill levels and allow for customized interventions. This study underscores the importance of bridging health literacy and eHealth literacy gaps in underserved communities to improve health outcomes and promote equitable access to health resources [69]. Future research endeavors should examine the effectiveness of educational interventions and digital health literacy programs to improve educational attainment and empower patients to make informed decisions about their health.

### Comparison With Prior Work

Compared to the studies conducted in Ethiopia (30.3%) [4] and Rwanda (14.3%) [75], more respondents at Donka Hospital had a high level of diabetes-related health literacy (35.6%). However, at Sanou Sourou Hospital in Burkina Faso, only 21.3% of the patients demonstrated adequate health literacy. Many participants obtained low health literacy scores, indicative of a deficiency in understanding and knowledge of health information. Health literacy scores exhibited an upward trend among individuals with higher levels of education. The correlation coefficient of 0.94 for education emphasizes the importance of this relationship, and the *P* value of <.001 supports its validity. Numerous studies have found significant correlations between health literacy and education; our results support this conclusion [76,77].

The positive correlation observed between education and health literacy suggests that endeavors aimed at enhancing education and literacy could have a significant impact on improving health literacy across both hospitals. Furthermore, the results from Sanou Sourou Hospital showed a moderately negative correlation between age and health literacy. The correlation coefficient of −0.336 indicates a propensity for health literacy to decrease with increasing age. This observation is consistent with the conclusions drawn in the study by Reisi et al [78], which reported a negative association between age and functional health literacy. The negative correlation between health literacy and age emphasizes the need for interventions tailored to the specific health literacy challenges of older populations.

Low levels of eHealth literacy were evident in both hospitals, indicating limited mastery of the use of digital technologies for health-related purposes. A study conducted by Shiferaw et al [79] in Ethiopia reported similarly low levels of internet use and eHealth literacy among patients with chronic illness in that setting. Consistent with findings regarding health literacy, education emerged as a significant predictor of eHealth literacy, with a higher level of education correlating with a higher level of eHealth literacy. This shows the importance of promoting digital health literacy through educational initiatives aimed at enhancing the utilization of digital technologies for health purposes. Notably, education exhibited the strongest positive correlation between health literacy and eHealth literacy in both hospitals. In Sanou Sourou Hospital, the correlation coefficient between eHEALS and education was 0.920, indicating a strong positive relationship between education and health literacy. This implies that individuals with higher levels of education exhibited correspondingly higher levels of health literacy. This result is consistent with the findings of Shiferaw et al [79], who found a 3.48-fold higher likelihood of high eHealth literacy among patients with a diploma or higher education level compared to those with primary education or lower education level [79]. Such consistency underscores the significance of educational interventions targeted at enhancing the overall level of education.

Age correlated negatively with health literacy and only weakly with eHealth literacy, emphasizing the need for interventions tailored to older populations. Previous studies have likewise demonstrated a negative correlation between age and eHealth literacy [80,81]. Conversely, neither gender nor income correlated significantly with health literacy or eHealth literacy. In a study by Norman and Skinner [41], men displayed a higher baseline level of eHealth literacy. Studies conducted by Meppelink et al [82] and Neufingerl et al [83] revealed significant correlations between income, gender, and eHealth literacy. Nonetheless, in line with the findings of Xesfingi and Vozikis [84], this study did not uncover a strong correlation between gender and eHealth literacy.

The results of the correlation analysis between the BHLS and eHEALS scores in this study are consistent with those of Monkman et al [71], suggesting that these instruments may capture different aspects of health literacy and may not consistently correlate within these populations. Targeted interventions and educational programs are needed to improve health literacy and eHealth literacy in both hospitals. Education and literacy promotion initiatives can serve as pivotal avenues for bolstering health literacy. Individual interventions and educational programs need to be developed to address the specific health literacy challenges encountered by older populations. In addition, concerted efforts should be directed toward improving digital health literacy to facilitate the effective utilization of digital technologies for health-related purposes.

### Implications for Practice and Research

This study emphasizes the urgent need to address the low level of health and eHealth literacy among the diabetic population in Burkina Faso and Guinea. Despite the widespread ownership of mobile phones, many people do not use internet services, which is a significant barrier to the effectiveness of eHealth solutions. To close this gap, it is essential to develop mHealth apps that also work offline and ensure access to health information regardless of the internet connection. In addition, the integration of voice interfaces into eHealth tools can improve usability for people with limited literacy skills, increasing participation and effectiveness. In addition to practical measures, policy measures to support the development and dissemination of these solutions are essential. Policymakers should allocate resources and create incentives to encourage the adoption of mHealth technologies tailored to the needs of underserved populations. In addition, partnerships between technology providers, health care organizations, and government agencies can facilitate the development and implementation of user-friendly eHealth solutions. By prioritizing eHealth literacy initiatives and integrating training into health care programs, stakeholders can empower people to use digital health resources effectively. Overall, these concerted efforts are critical to closing the health literacy gap and ensuring equitable access to digital health resources for all people, especially those in underserved communities.

### Limitations

The study was constrained by several limitations. First, the small sample size, limited to 2 hospitals in Guinea and Burkina Faso, restricts the generalizability of findings to other regions in sub-Saharan Africa. Additionally, relying on self-reported health literacy and eHealth literacy introduces potential biases, with participants possibly overestimating their skills. The cross-sectional design offers only a snapshot of health literacy levels at a single point, lacking information on changes over time. Moreover, the study solely used the eHEALS and BHLS scales, potentially missing nuances in health literacy and eHealth literacy complexity.

Despite these constraints, the study offers valuable insights. It underscores the need for future research with larger sample sizes, broader geographic representation, comprehensive assessment tools, longitudinal designs, and attention to language barriers.

### Conclusion

In analyzing data from Donka and Sanou Sourou hospitals, significant disparities in health and eHealth literacy were uncovered, underscoring the urgent need for targeted interventions. Education emerged as a key determinant of literacy levels, highlighting the importance of educational initiatives. Tailored interventions for older adult populations are imperative, given the negative correlation between age and health literacy. Although gender and income showed no significant correlation with literacy, the multifaceted nature of health literacy warrants comprehensive interventions. Prioritizing educational programs and digital literacy initiatives can empower individuals and foster better health outcomes in Burkina Faso and Guinea.

## Abbreviations

BHLS: Brief Health Literacy Screen
eHEALS: eHealth Literacy Scale
mHealth: mobile health
Ophelia: Optimising Health Literacy and Access
